# Histogram and Vertical Bar diagram: Often Misapprehended Concept

**DOI:** 10.1007/s10557-014-6529-6

**Published:** 2014-06-13

**Authors:** Mageshwaran Lakshmanan

**Affiliations:** Jawaharlal Institute of Postgraduate Medical Education & Research, Puducherry, Pondicherry, India

Dear editor,

The inhibition of platelet activation by clopidogrel was shown to prevent hypertension induced cardiac inflammation and fibrosis in mice by Jia et al. and was published recently [1].

We fail to understand why authors have specified all the graphical representation of data in the above mentioned study as histogram. Any data can be classified into categorical and continuous data. For categorical data, one of the most commonly used graphical representations is bar diagram or bar charts. It can be shown either in horizontal or vertical direction. In vertical bar chart, the X-axis comprises of different groups of data as bars and Y-axis represents its value as the height of the bars. All the bars are equally spaced in X- axis and the spacing between the bars indicates that data are categorical, unique and no way related to each other [2].

However, this is not for the case of histogram. A histogram is usually used for the representation of the distribution of continuous data. Histogram, also like vertical bar chart, has bars. Even though histogram closely resembles vertical bar diagram in its image, the spacing between the bars is absent. This is because, the data used are continuous as a spectrum of light and lines of the bars denote a particular class interval. Hence all the bars are drawn side by side and there are no gaps between the bars in the histogram [2].

With the increased use of modern statistical programmes, the distinction between these two graphical data representation is often lost. In the above mentioned study, authors have studied four groups of mice receiving different drugs (vehicle, clopidogrel, angiotensin II and clopidogrel + angiotensin II) and estimated the mRNA levels of several cytokines, blood pressure and cardiac myocyte size. This data belong to the categorical type and hence bar chart should be used ideally. Even though bar chart was shown in the figure, it was stated as histogram in the footnote. This might be a trivial issue, but it gains significance in the statistical point of view about proper usage and mentioning of graphical representation of data.

The standard guidelines for reporting the animal studies mandate the mentioning of various dose of all the drugs used in a particular study [3]. The authors studied the effect of angiotensin II induced hypertension, its effect on cardiac inflammation and its relation with clopidogrel treatment. The angiotensin II was administered at the dose of 1,500 ng/Kg/day subcutaneously for seven days. The authors also studied the acute effect of hypertension by angiotensin II on platelet activation in different mice groups. Nevertheless, the dose of angiotensin II used for latter study group was not mentioned in the study. If the same dose were used, it should have been mentioned to avoid unwanted misunderstanding.

Moreover, it is a good practice in research to administer an investigating drug in pure chemical form. When an investigating drug in the form of tablet is crushed and administered by gavage, the possibility of confounding effect by excipient could not be efficiently eliminated. Also a uniform drug delivery and absorption will not be ensured. The authors have not given any proper justification for using the clopidogrel tablets in the study.

Despite these slips, the article in our opinion did contribute to understanding the role of platelets in angiotensin II induced cardiac inflammation.
